# Supplementary material for: Synthesis of Novel α-Trifluorothioanisole Derivatives Containing Phenylpyridine Moieties with Herbicidal Activity
Source: Molecules. 2022 Sep 10;27(18):5879. doi: 10.3390/molecules27185879 (PMC9505602; doi:10.3390/molecules27185879)
Supplement: Supplementary file 1 [file molecules-27-05879-s001.zip › molecules-1902475-supplementary.pdf]

Article

Synthesis of Novel α-Trifluorothioanisole Derivatives Containing Phenylpyridine Moieties with Herbicidal Activity

Zengfei Cai [1], Wenliang Zhang [1], Zhongjie Yan [2] and Xiaohua Du [1,*]

[1] Catalytic Hydrogenation Research Center, Zhejiang Key Laboratory of Green Pesticides and Cleaner Production Technology, Zhejiang Green Pesticide Collaborative Innovation Center, Zhejiang University of Technology, Hangzhou 310014, P. R. China
[2] Agrowin(Ningbo) bioscience Co., Ltd., Ningbo, 315100, China.
* Correspondence: duxiaohua@zjut.edu.cn (X.-H.D.)

Supporting Information

Contents

1. Data of 5a in Single-crystal X-ray Diffraction 2

2. [1] H NMR spectra and ^{13}C NMR spectra of target compounds 5a-5f, 6a-6f, and 7a-7f.... 5

1. Data of 5a in Single-crystal X-ray Diffraction

Table S1. Crystal data and structure refinement parameters of compound **5a**.

Parameter	Value
Empirical formula	$C_{20}H_{12}ClF_6NOS$
Formula weight	463.82
Temperature	296 K
Wavelength	1.34139 Å
Crystal system	Triclinic
Space group	*P*-1
Unit cell dimensions	a = 4.9349(3) Å α = 82.102(3)°<br>b = 8.7426(6) Å β = 89.490(3)°<br>c = 23.4328(14) Å γ = 78.286(3)°
Volume	980.35(11) $Å^3$
Z	2
Density (calculated)	1.571 Mg/m^3
Absorption coefficient	2.183 mm^{-1}
F(000)	468
Crystal size	0.16 × 0.12 × 0.08 mm^3
Theta range for data collection	3.314 to 55.348°
Index ranges	$-6 \leq h \leq 6$, $-8 \leq k \leq 10$, $-28 \leq l \leq 28$
Reflections collected	12877
Independent reflections	3689 [R(int) = 0.0604]
Completeness to theta = 53.594°	99.3%
Absorption correction	Semi-empirical from equivalents
Max. and min. transmission	0.7508 and 0.3676
Refinement method	Full-matrix least-squares on F^2
Data / restraints / parameters	3689 / 0 / 271
Goodness-of-fit on F^2	1.081
Final *R* indices [$I > 2$sigma(I)]	R_1 = 0.0616, ωR_2 = 0.1787
R indices (all data)	R_1 = 0.0718, ωR_2 = 0.1900
Extinction coefficient	None
Largest diff. peak and hole	0.549 and -0.369 e.$Å^{-3}$

Crystal structure determination of 5a

Crystal Data. $C_{20}H_{12}ClF_6NOS$ (M =463.82 g/mol): triclinic, space group P-1, a = 4.9349(3) Å, b = 8.7426(6) Å, c = 23.4328(14) Å, β = 89.490(3)°, V = 980.35(11) $Å^3$, Z = 2, T = 298 K, $Dcalc$ = 1.571 Mg/m^3, 12877 reflections measured ($3.314° \leq 2\theta \leq 55.348°$), 3689 unique ($R_{int}$ = 0.0604) which were used in all calculations. The final R_1 was 0.0616 ($I > 2\sigma(I)$) and wR_2 was 0.1787 (all data).

Table S2. Bond angles of compound **5a**.

Atoms	Angle (°)	Atoms	Angle (°)
C(20)-S(1)-C(17)	100.49(14)	C(10)-C(11)-H(11)	119.9
C(10)-O(1)-C(13)	118.2(2)	C(12)-C(11)-C(10)	120.2(3)
C(1)-N(1)-C(5)	119.5(2)	C(12)-C(11)-H(11)	119.9
N(1)-C(1)-H(1)	118.3	C(7)-C(12)-H(12)	119.6
N(1)-C(1)-C(2)	123.4(3)	C(11)-C(12)-C(7)	120.8(3)
C(2)-C(1)-H(1)	118.3	C(11)-C(12)-H(12)	119.6
C(1)-C(2)-C(3)	118.3(2)	O(1)-C(13)-H(13A)	109.7
C(1)-C(2)-C(6)	121.4(3)	O(1)-C(13)-H(13B)	109.7
C(3)-C(2)-C(6)	120.3(3)	O(1)-C(13)-C(14)	109.8(2)
C(2)-C(3)-H(3)	120.8	H(13A)-C(13)-H(13B)	108.2
C(4)-C(3)-C(2)	118.5(3)	C(14)-C(13)-H(13A)	109.7
C(4)-C(3)-H(3)	120.8	C(14)-C(13)-H(13B)	109.7
C(3)-C(4)-Cl(1)	116.3(2)	C(15)-C(14)-C(13)	122.0(3)
C(3)-C(4)-C(5)	120.8(2)	C(19)-C(14)-C(13)	119.3(3)

C(5)-C(4)-Cl(1)	122.82(19)	C(19)-C(14)-C(15)	118.7(3)
N(1)-C(5)-C(4)	119.5(2)	C(14)-C(15)-H(15)	119.7
N(1)-C(5)-C(7)	114.7(2)	C(16)-C(15)-C(14)	120.5(3)
C(4)-C(5)-C(7)	125.8(2)	C(16)-C(15)-H(15)	119.7
F(1)-C(6)-F(2)	107.5(3)	C(15)-C(16)-H(16)	120.3
F(1)-C(6)-F(3)	107.8(3)	C(17)-C(16)-C(15)	119.3(3)
F(1)-C(6)-C(2)	113.0(3)	C(17)-C(16)-H(16)	120.3
F(2)-C(6)-C(2)	111.9(3)	C(16)-C(17)-S(1)	119.0(2)
F(3)-C(6)-F(2)	103.9(3)	C(18)-C(17)-S(1)	120.9(2)
F(3)-C(6)-C(2)	112.2(3)	C(18)-C(17)-C(16)	120.1(3)
C(8)-C(7)-C(5)	120.0(2)	C(17)-C(18)-H(18)	120
C(8)-C(7)-C(12)	117.9(2)	C(19)-C(18)-C(17)	120.1(3)
C(12)-C(7)-C(5)	122.2(2)	C(19)-C(18)-H(18)	120
C(7)-C(8)-H(8)	119	C(14)-C(19)-H(19)	119.4
C(7)-C(8)-C(9)	122.0(3)	C(18)-C(19)-C(14)	121.3(3)
C(9)-C(8)-H(8)	119	C(18)-C(19)-H(19)	119.4
C(8)-C(9)-H(9)	120.6	F(4)-C(20)-S(1)	115.1(3)
C(10)-C(9)-C(8)	118.8(3)	F(4)-C(20)-F(5)	106.8(3)
C(10)-C(9)-H(9)	120.6	F(4)-C(20)-F(6)	103.1(3)
O(1)-C(10)-C(9)	124.6(3)	F(5)-C(20)-S(1)	108.8(3)
O(1)-C(10)-C(11)	115.0(2)	F(5)-C(20)-F(6)	107.8(3)
C(11)-C(10)-C(9)	120.4(2)	F(6)-C(20)-S(1)	114.8(2)

Table S3. Atomic coordinates (× 10^4) and equivalent isotropic displacement parameters (Å^2 × 10^3) of the nonhydrogen atoms.

Atom	*x*	*y*	*z*	U_{eq}
Cl (1)	4881(2)	3250(1)	2604(2)	101(1)
Cl(1)	-1097(2)	3964(1)	9436(1)	65(1)
S(1)	17002(2)	-2331(1)	6020(1)	80(1)
F(1)	-8858(6)	8902(3)	9528(1)	138(1)
F(2)	-6219(5)	10543(3)	9363(1)	106(1)
F(3)	-9021(5)	10381(3)	8726(1)	110(1)
F(4)	13799(5)	-4278(3)	5864(1)	104(1)
F(5)	17528(6)	-4524(3)	5407(2)	129(1)
F(6)	14065(7)	-2681(3)	5144(1)	118(1)
O(1)	6881(4)	2066(2)	7347(1)	67(1)
N(1)	-2314(5)	7505(3)	8130(1)	63(1)
C(1)	-4263(7)	8538(3)	8350(1)	64(1)
C(2)	-5204(5)	8292(3)	8902(1)	54(1)
C(3)	-4115(5)	6879(3)	9243(1)	55(1)
C(4)	-2139(5)	5791(3)	9012(1)	49(1)
C(5)	-1198(5)	6125(3)	8455(1)	48(1)
C(6)	-7342(7)	9509(3)	9134(2)	69(1)
C(7)	909(5)	5055(3)	8160(1)	48(1)
C(8)	682(6)	5047(3)	7575(1)	57(1)
C(9)	2609(6)	4070(3)	7280(1)	58(1)
C(10)	4852(5)	3100(3)	7584(1)	54(1)
C(11)	5154(6)	3106(3)	8168(1)	58(1)
C(12)	3207(5)	4063(3)	8456(1)	54(1)
C(13)	6923(6)	2141(4)	6750(1)	66(1)
C(14)	9411(5)	999(3)	6577(1)	56(1)
C(15)	10724(6)	-304(4)	6954(1)	65(1)
C(16)	12987(6)	-1345(4)	6779(1)	65(1)
C(17)	13958(5)	-1060(3)	6226(1)	56(1)
C(18)	12648(7)	226(4)	5852(1)	67(1)
C(19)	10402(7)	1238(4)	6028(1)	65(1)
C(20)	15528(7)	-3485(4)	5595(2)	70(1)

Table S4. Hydrogen coordinates (× 10⁴) and isotropic displacement parameters (Å² × 10³).

Atom	*x*	*y*	*z*	U_{eq}
H (1)	467	3247	-1520	110
H(1)	-5044	9495	8114	77
H(3)	-4715	6665	9626	66
H(8)	-839	5730	7366	68
H(9)	2393	4067	6878	69
H(11)	6711	2448	8373	70
H(12)	3421	4051	8859	65
H(13A)	7000	3226	6572	79
H(13B)	5210	1877	6611	79
H(15)	10067	-484	7335	79
H(16)	13861	-2244	7035	79
H(18)	13300	411	5471	81
H(19)	9512	2122	5767	78

Table S5. Bond lengths of compound **5a**.

Atoms	Length (Å)	Atoms	Length (Å)
Cl(1)-C(4)	1.744(2)	C(7)-C(12)	1.402(4)
S(1)-C(17)	1.783(3)	C(8)-H(8)	0.95
S(1)-C(20)	1.768(4)	C(8)-C(9)	1.389(4)
F(1)-C(6)	1.305(4)	C(9)-H(9)	0.95
F(2)-C(6)	1.324(4)	C(9)-C(10)	1.385(4)
F(3)-C(6)	1.319(4)	C(10)-C(11)	1.379(4)
F(4)-C(20)	1.310(4)	C(11)-H(11)	0.95
F(5)-C(20)	1.316(4)	C(11)-C(12)	1.376(4)
F(6)-C(20)	1.317(4)	C(12)-H(12)	0.95
O(1)-C(10)	1.374(3)	C(13)-H(13A)	0.99
O(1)-C(13)	1.392(4)	C(13)-H(13B)	0.99
N(1)-C(1)	1.331(4)	C(13)-C(14)	1.508(4)
N(1)-C(5)	1.350(3)	C(14)-C(15)	1.389(4)
C(1)-H(1)	0.95	C(14)-C(19)	1.376(4)
C(1)-C(2)	1.373(4)	C(15)-H(15)	0.95
C(2)-C(3)	1.385(4)	C(15)-C(16)	1.388(4)
C(2)-C(6)	1.495(4)	C(16)-H(16)	0.95
C(3)-H(3)	0.95	C(16)-C(17)	1.385(4)
C(3)-C(4)	1.380(3)	C(17)-C(18)	1.375(4)
C(4)-C(5)	1.395(4)	C(18)-H(18)	0.95
C(5)-C(7)	1.484(3)	C(18)-C(19)	1.370(4)
C(7)-C(8)	1.379(4)	C(19)-H(19)	0.95

2. ¹H NMR and ¹³C NMR spectra of target compounds 5a-5f, 6a-6f, and 7a-7f

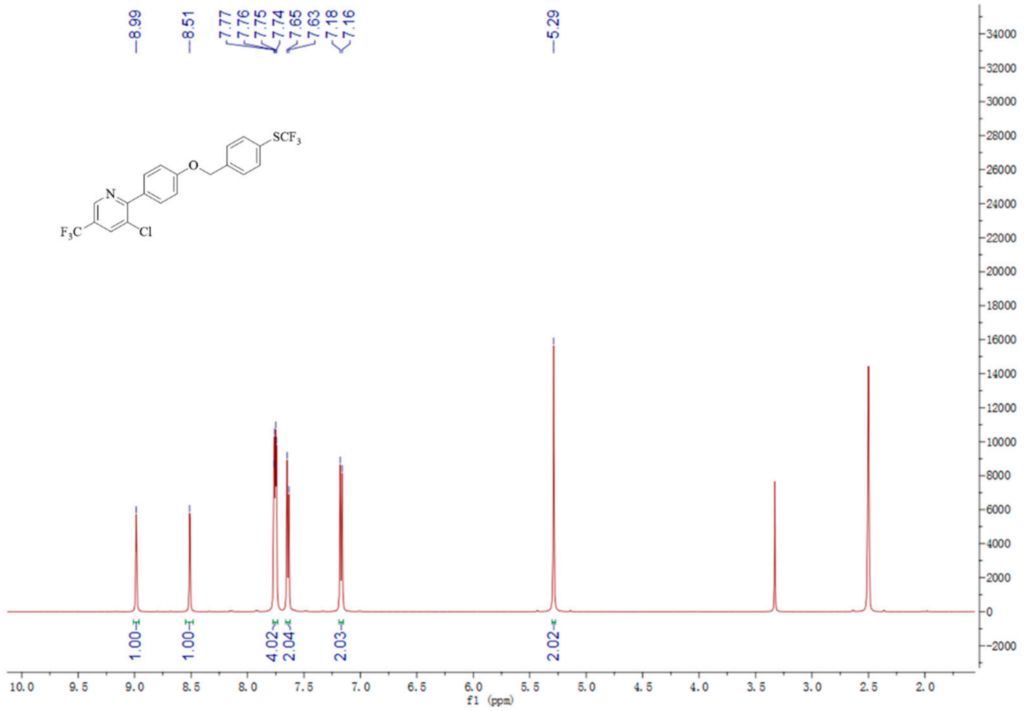

Figure S1. The ¹H NMR spectrum of **5a.**

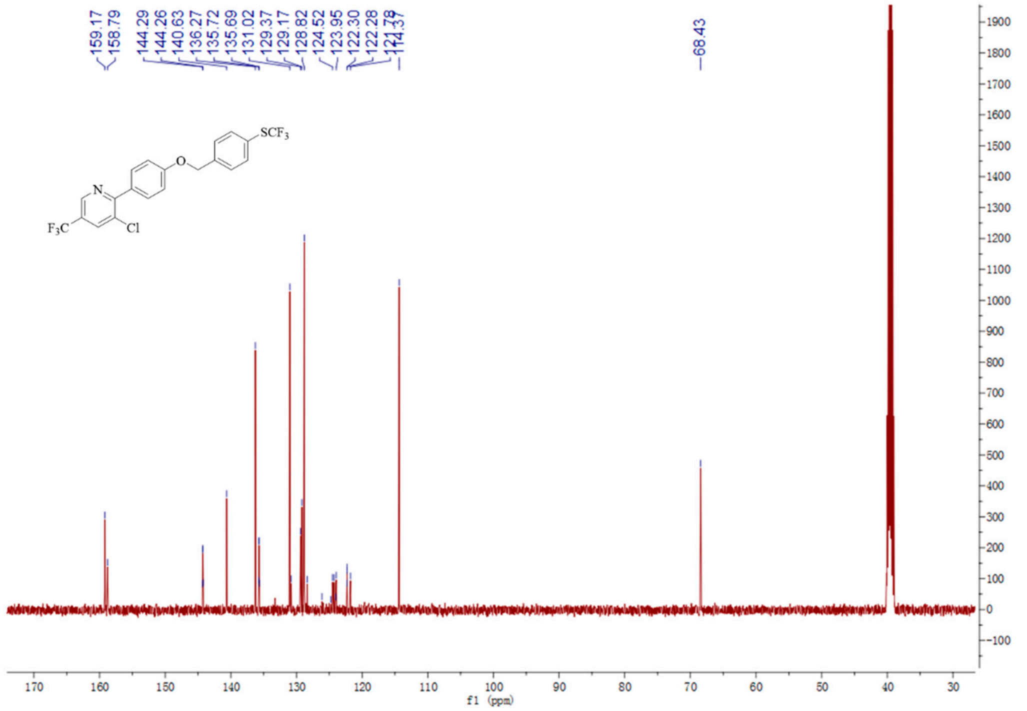

Figure S2. The ¹³C NMR spectrum of **5a.**

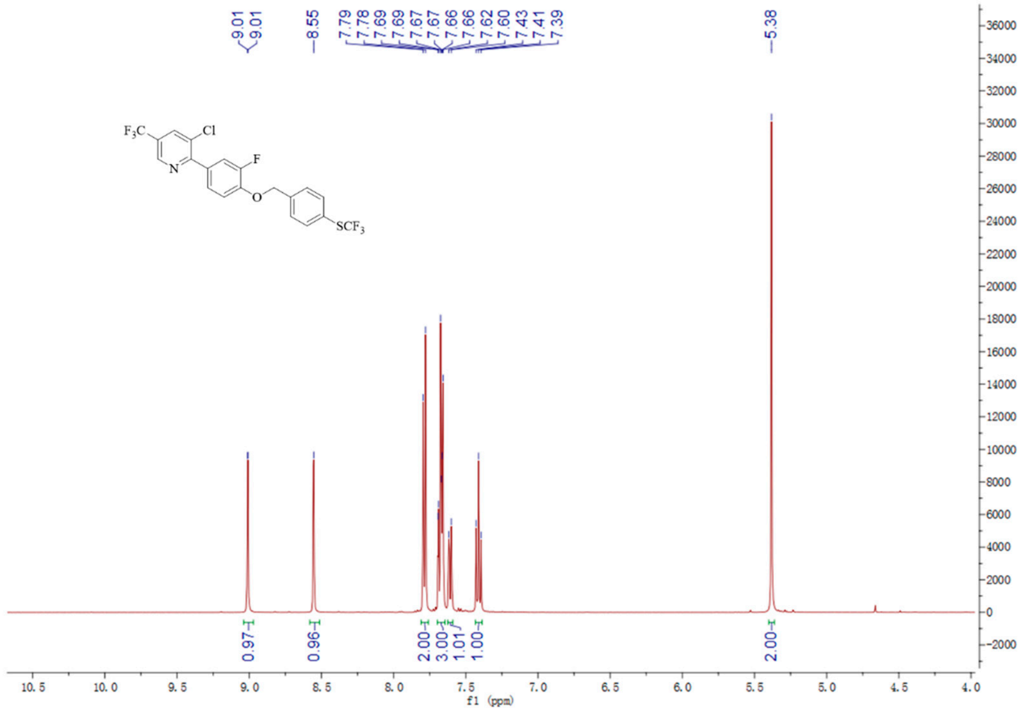

Figure S3. The ^{1}H NMR spectrum of **5b.**

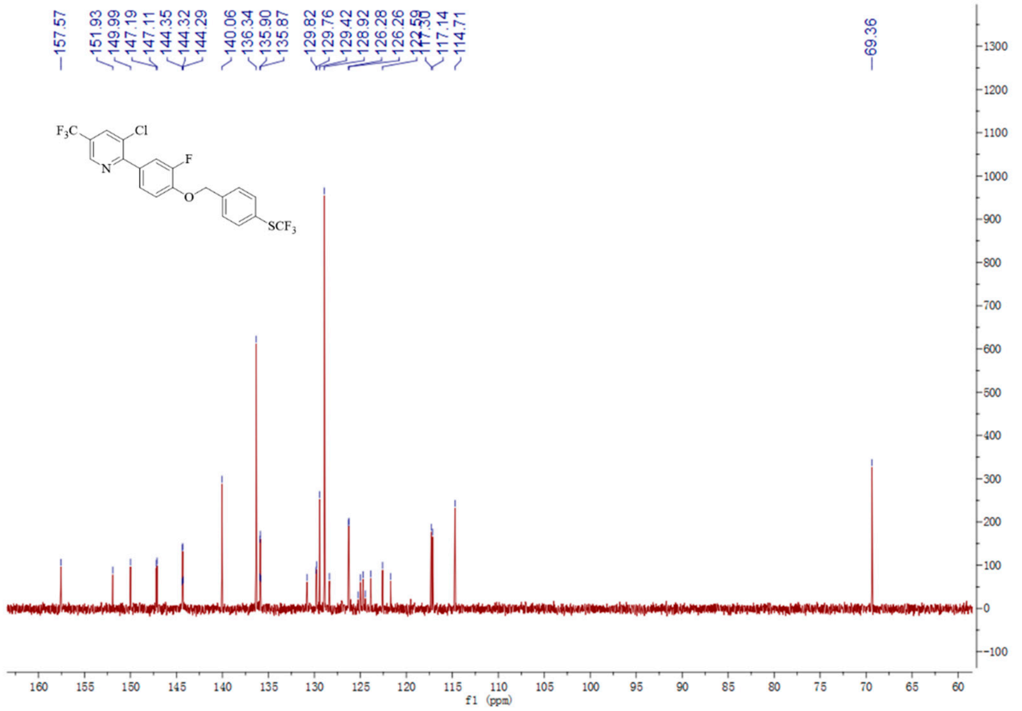

Figure S4. The ^{13}C NMR spectrum of **5b.**

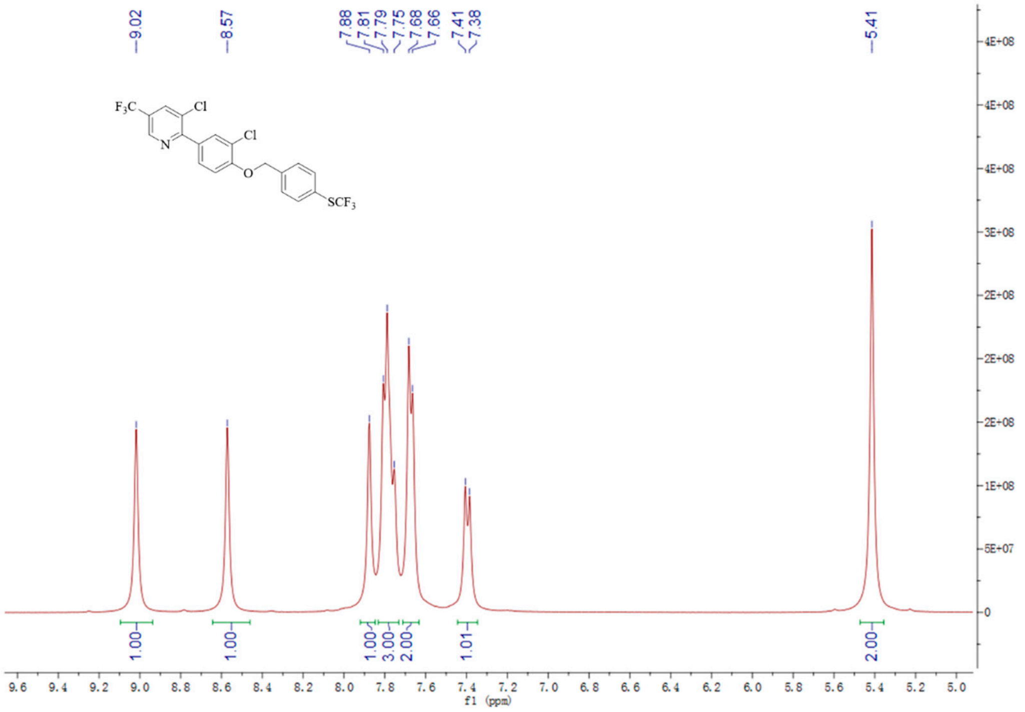

Figure S5. The ^{1}H NMR spectrum of **5c.**

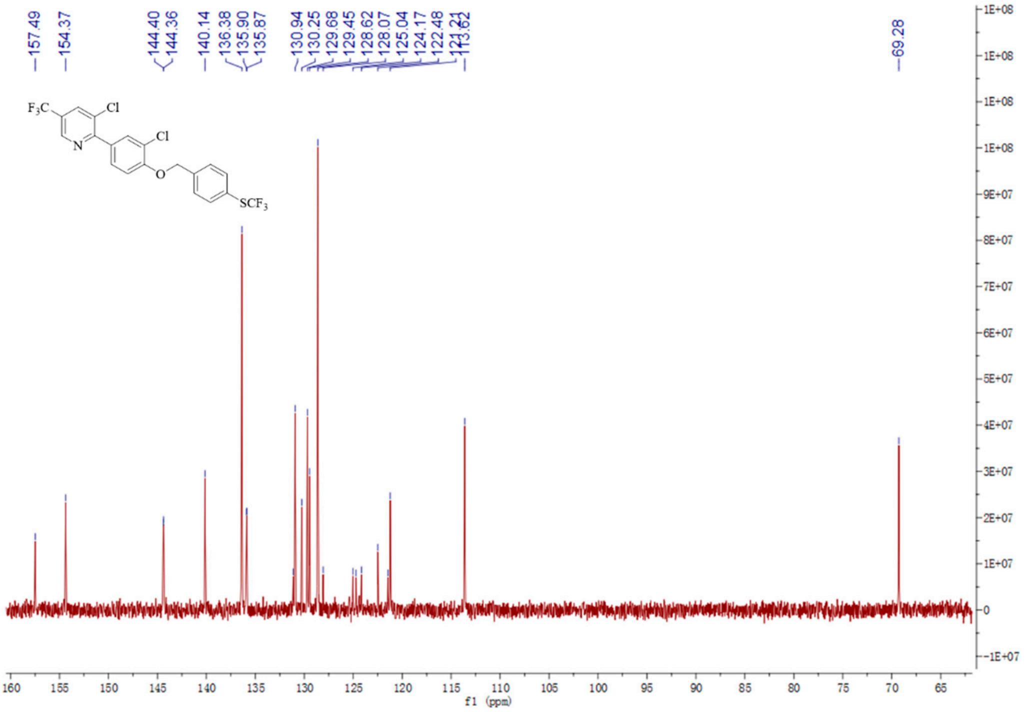

Figure S6. The ^{13}C NMR spectrum of **5c.**

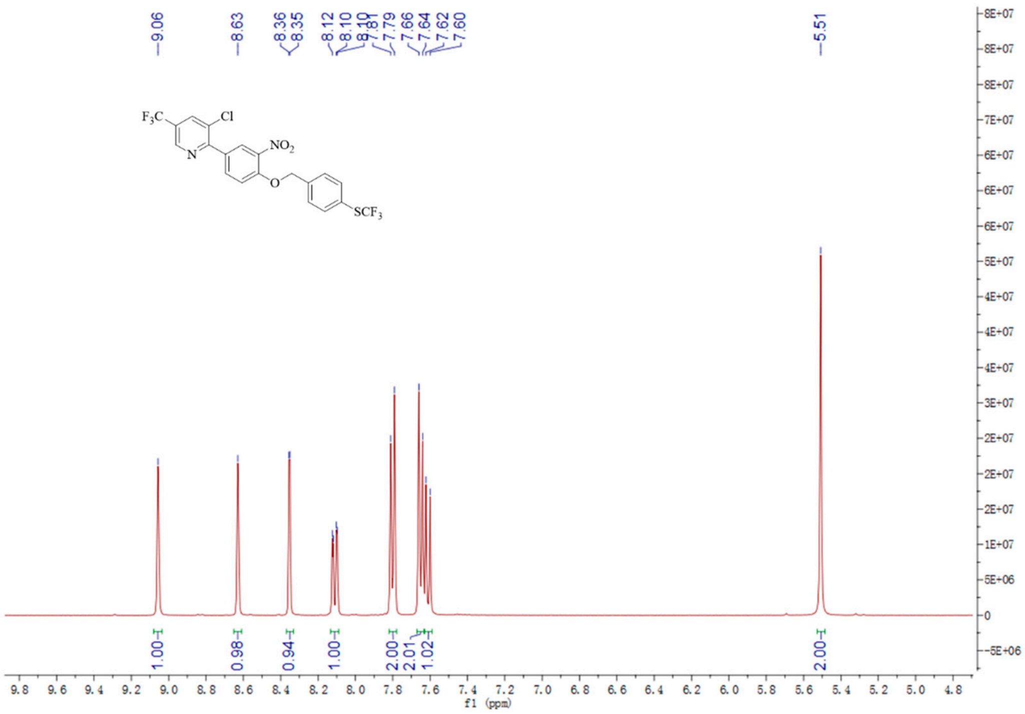

Figure S7. The ^{1}H NMR spectrum of **5d.**

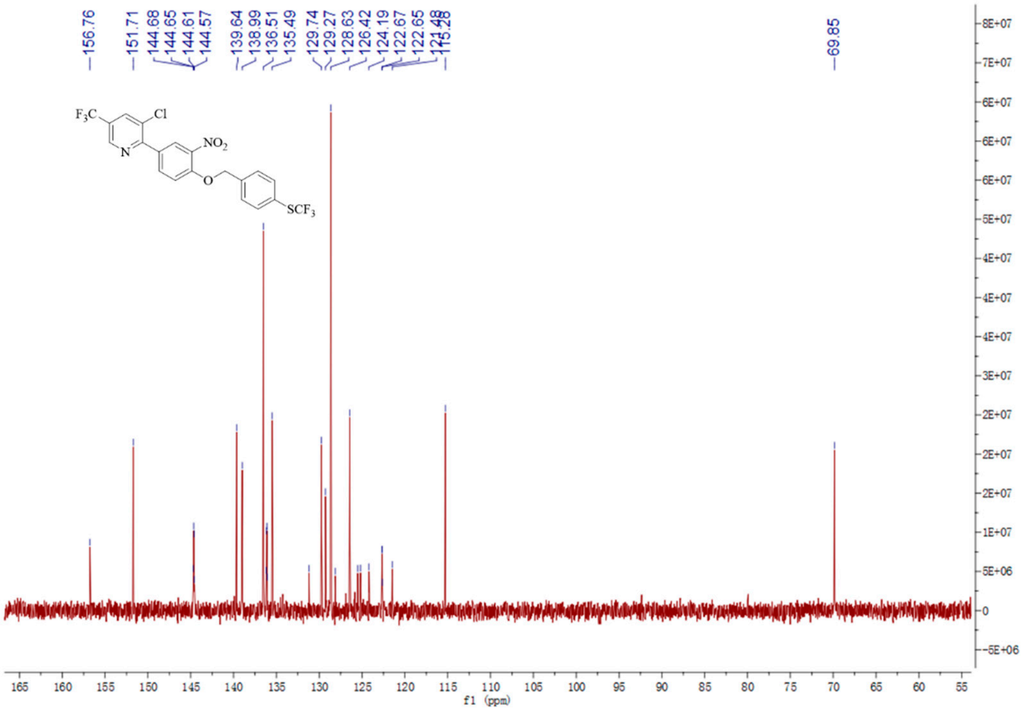

Figure S8. The ^{13}C NMR spectrum of **5d.**

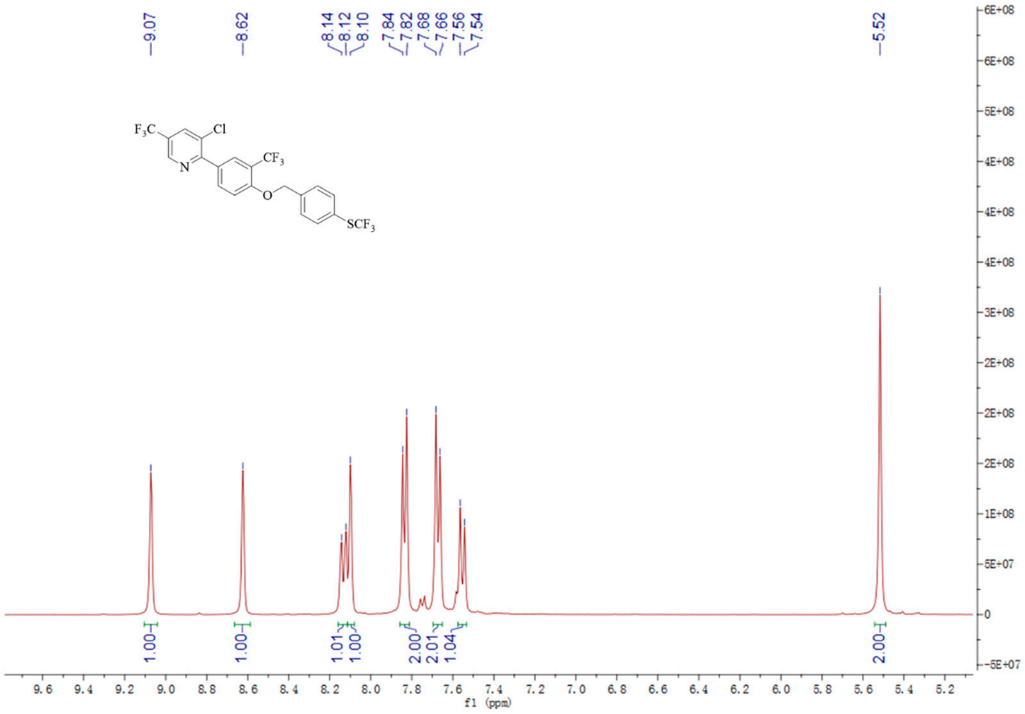

Figure S9. The ^{1}H NMR spectrum of **5e.**

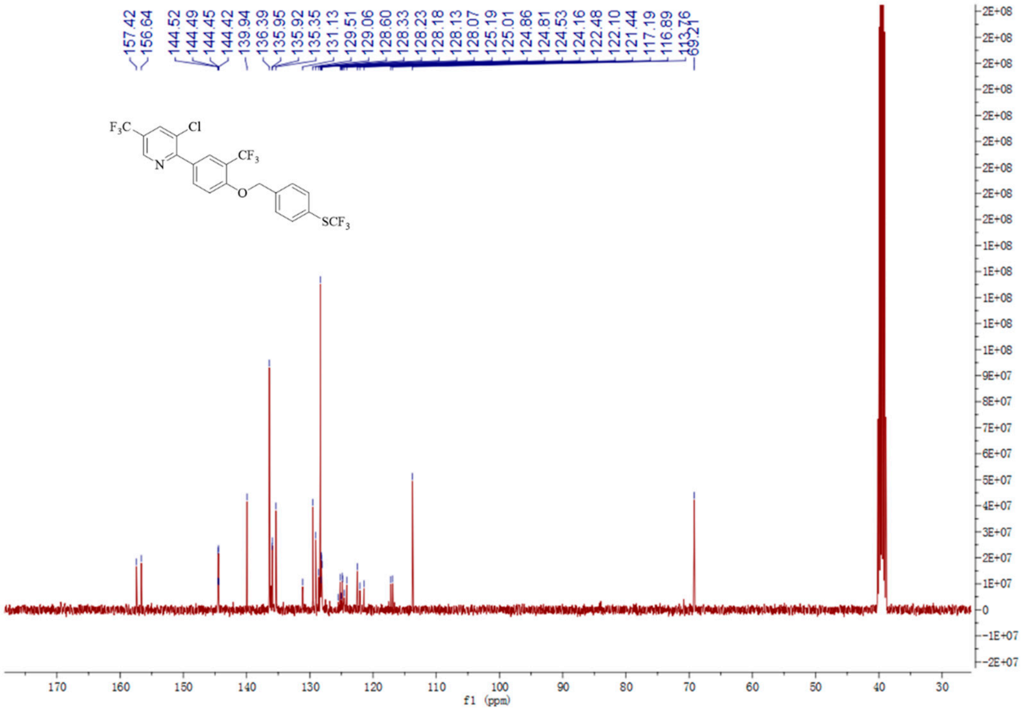

Figure S10. The ^{13}C NMR spectrum of **5e.**

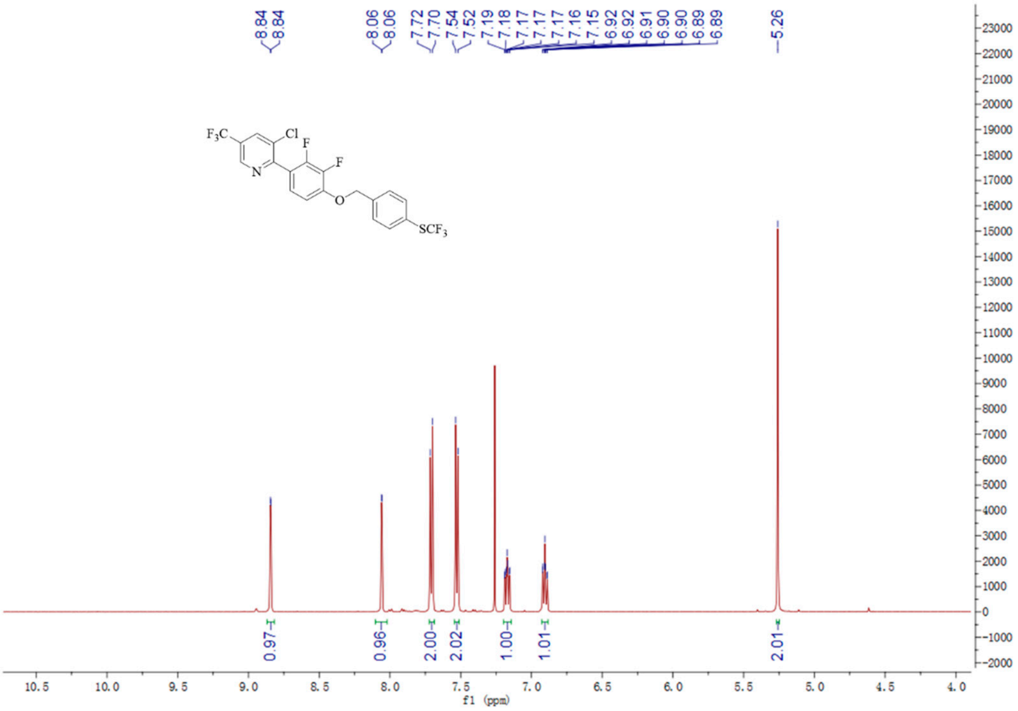

Figure S11. The ^{1}H NMR spectrum of **5f.**

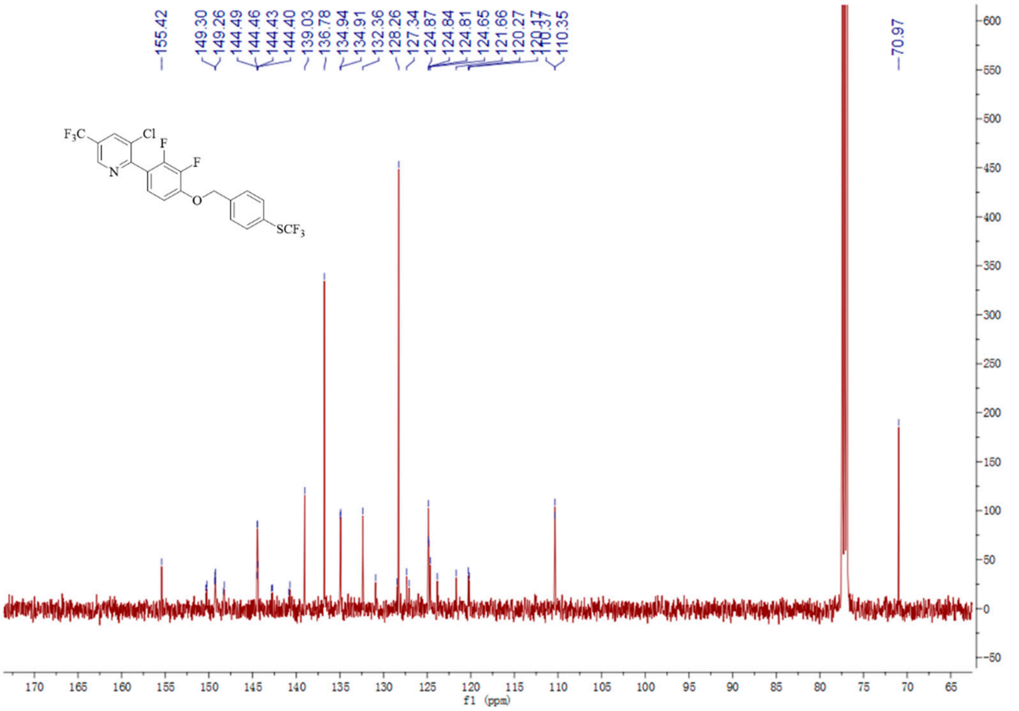

Figure S12. The ^{13}C NMR spectrum of **5f.**

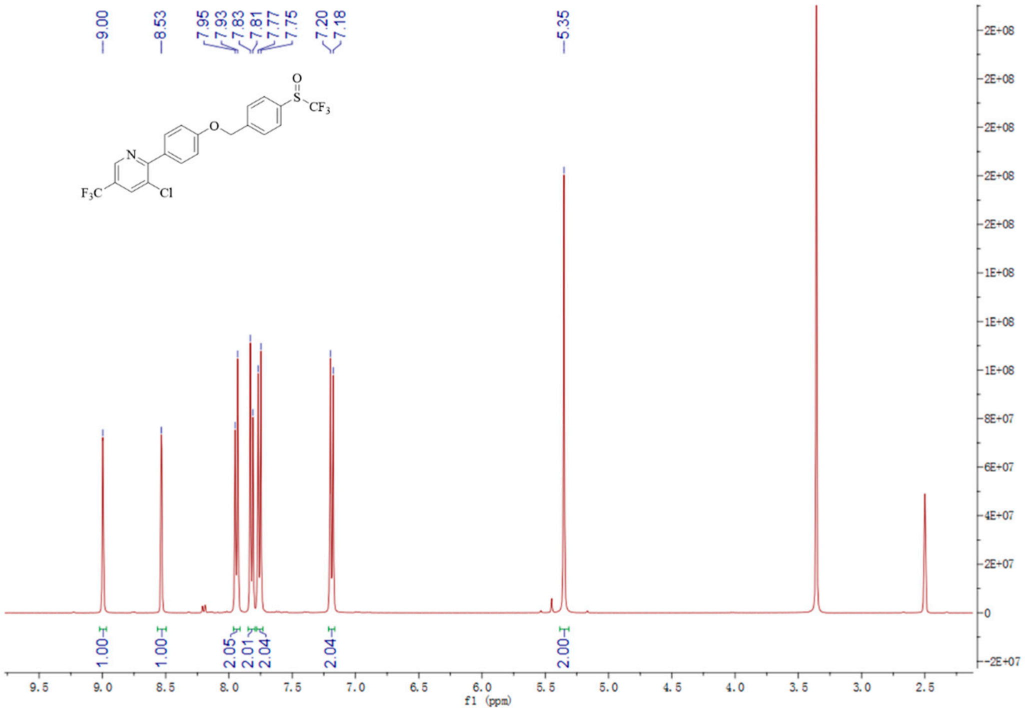

Figure S13. The ^{1}H NMR spectrum of **6a.**

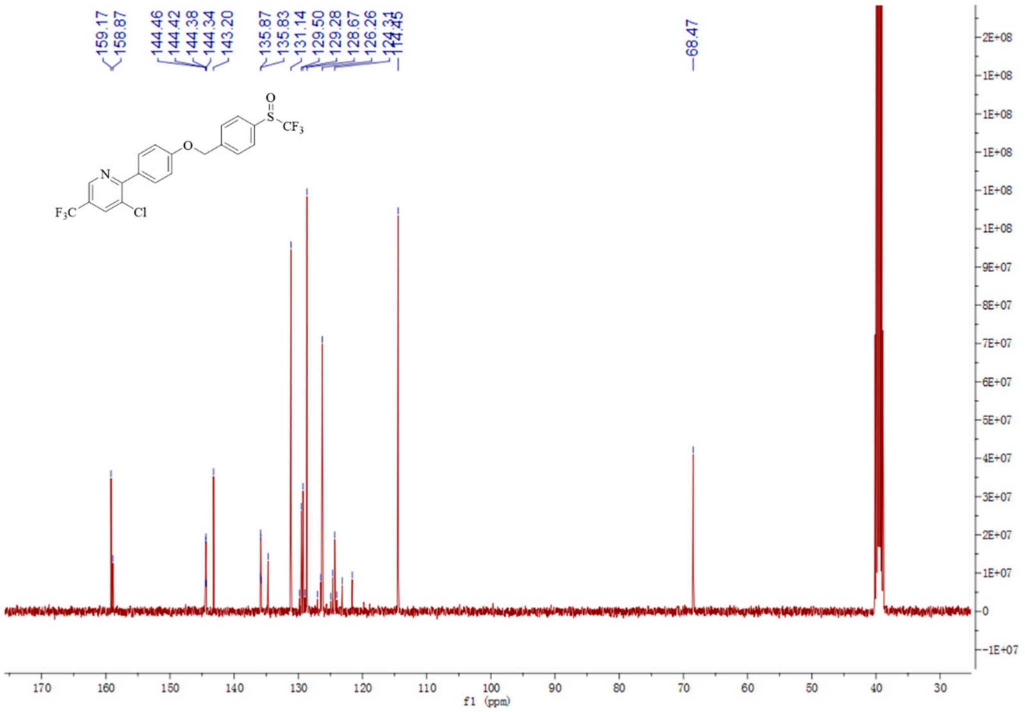

Figure S14. The ^{13}C NMR spectrum of **6a.**

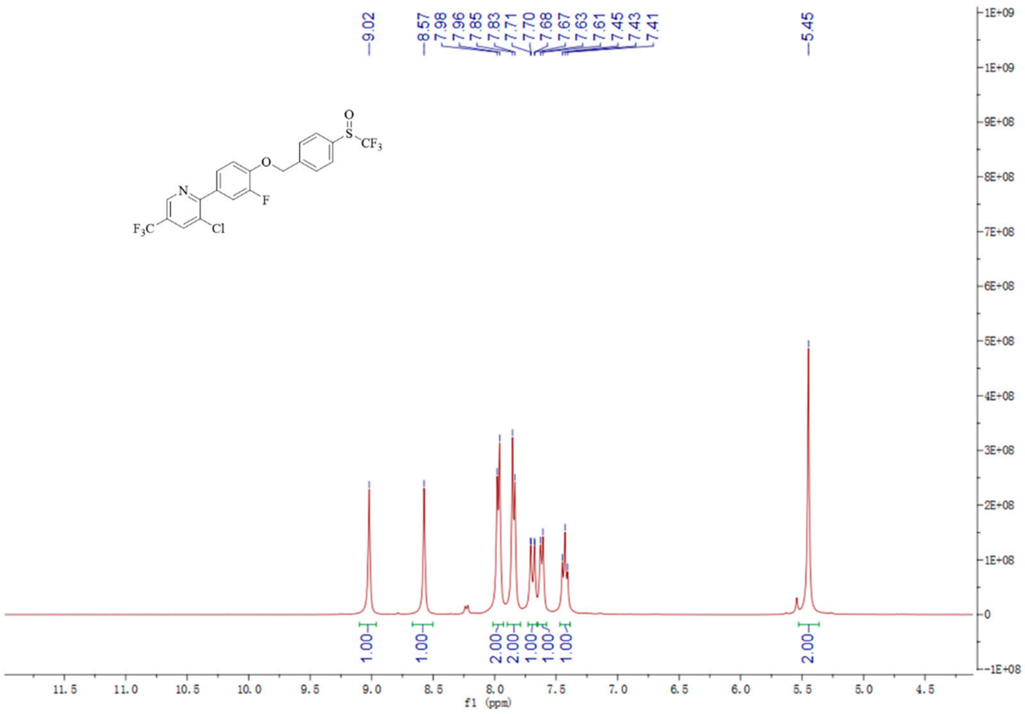

Figure S15. The ^{1}H NMR spectrum of **6b.**

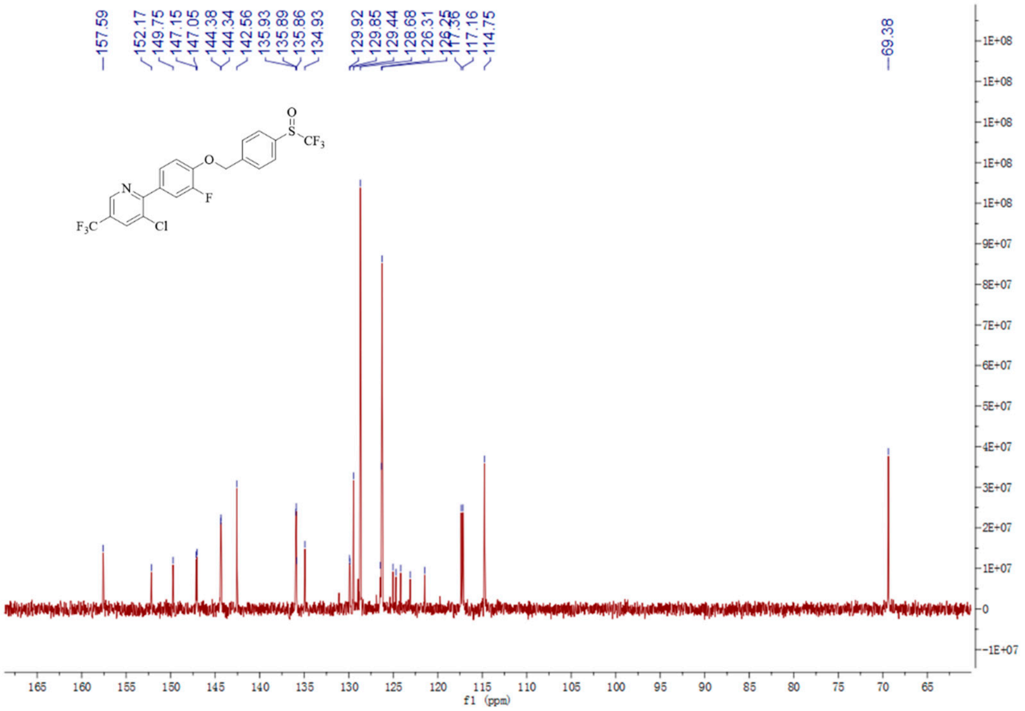

Figure S16. The ^{13}C NMR spectrum of **6b.**

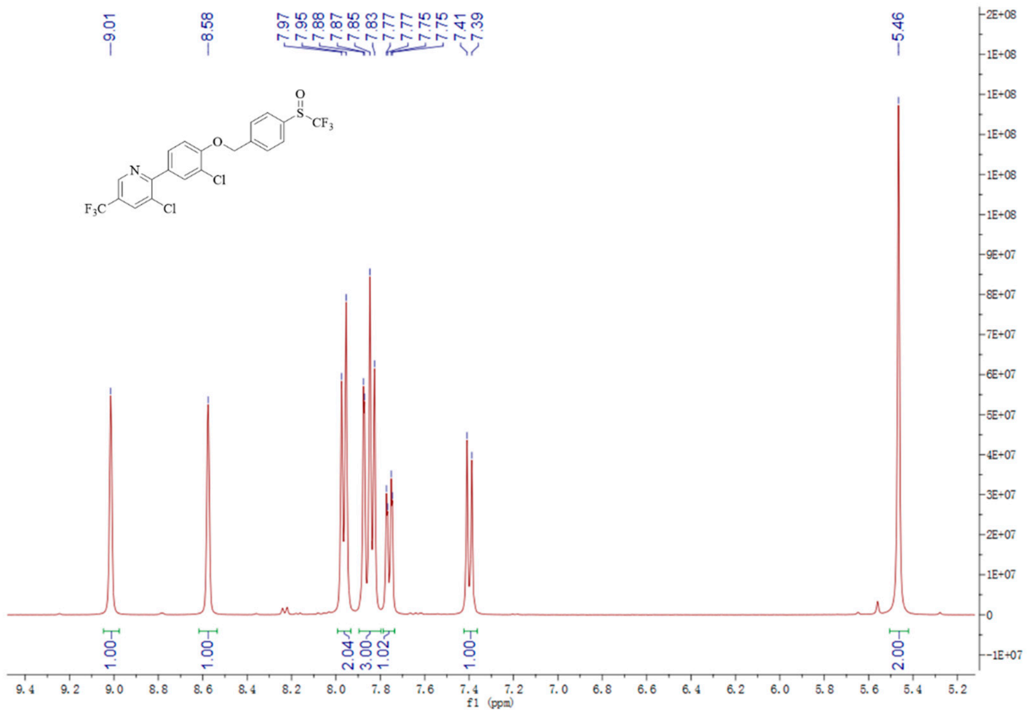

Figure S17. The ^{1}H NMR spectrum of **6c.**

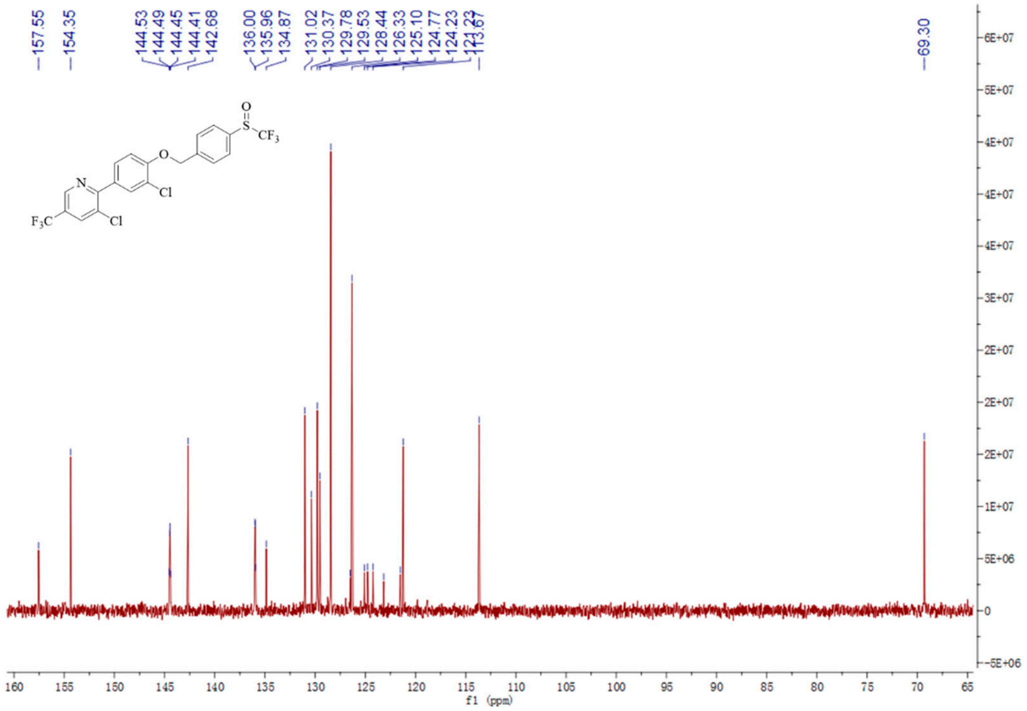

Figure S18. The ^{13}C NMR spectrum of **6c.**

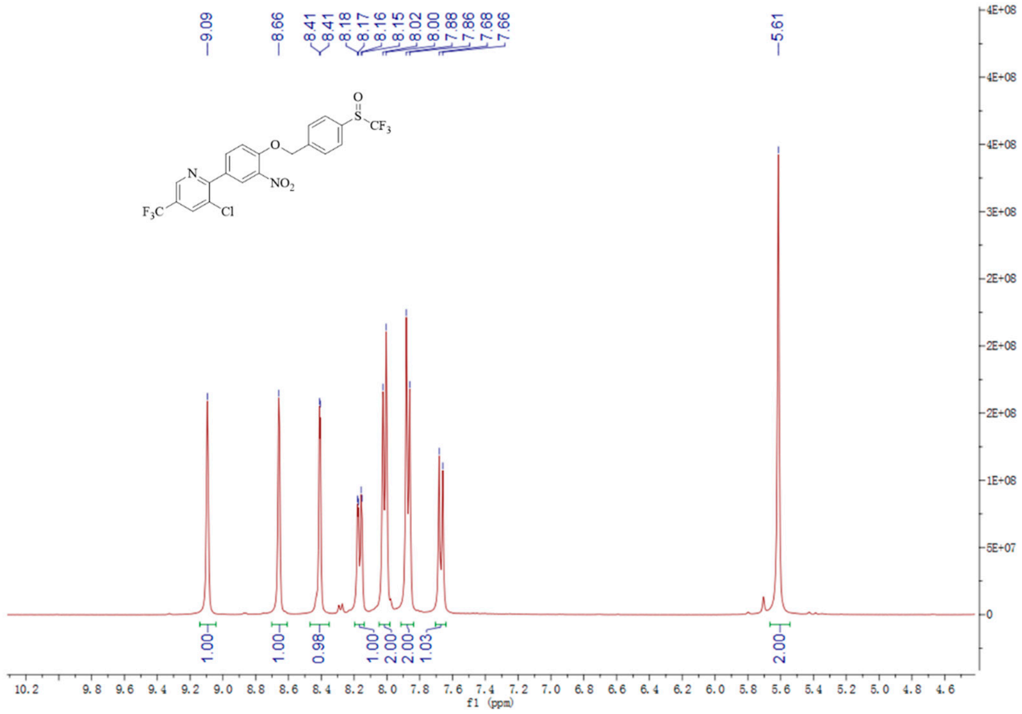

Figure S19. The ^{1}H NMR spectrum of **6d.**

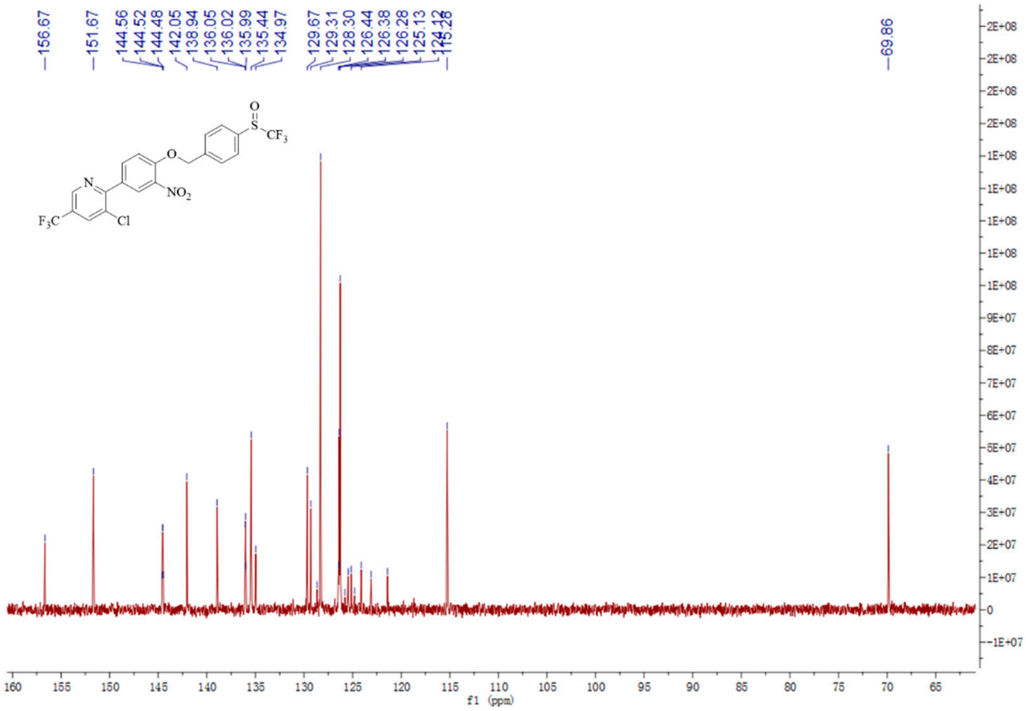

Figure S20. The ^{13}C NMR spectrum of **6d.**

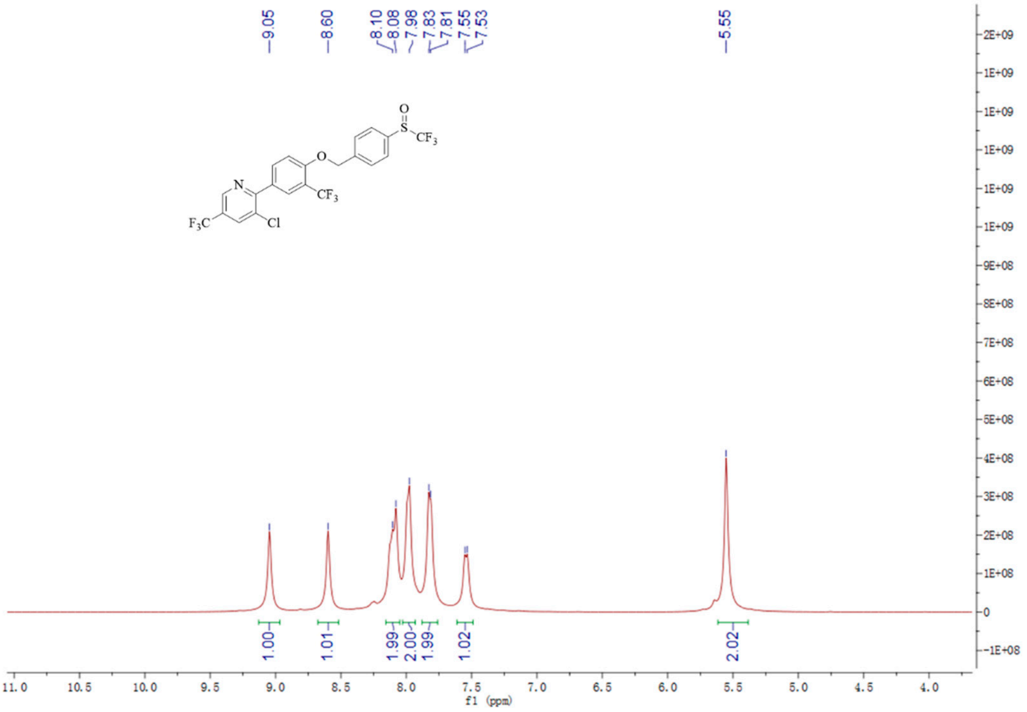

Figure S21. The ^{1}H NMR spectrum of **6e.**

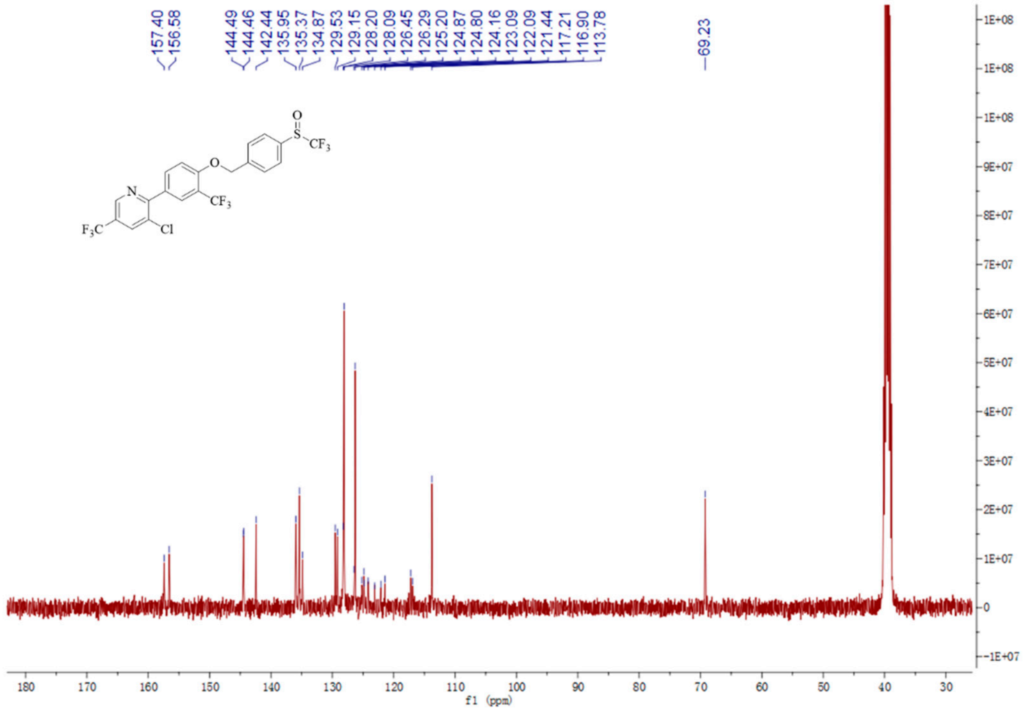

Figure S22. The ^{13}C NMR spectrum of **6e.**

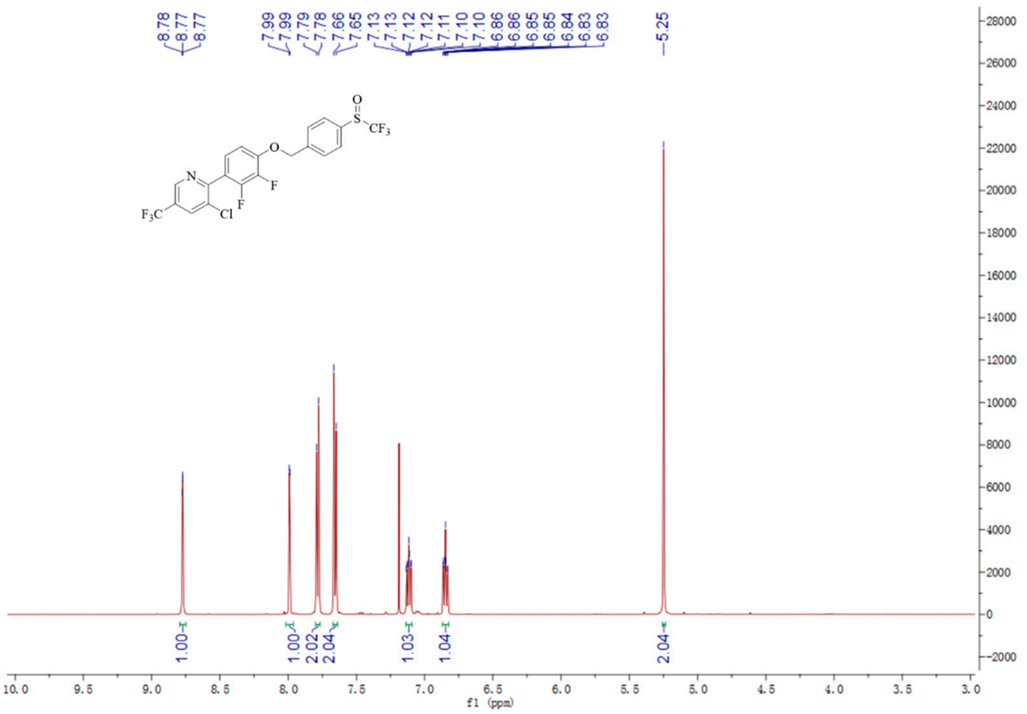

Figure S23. The ^{1}H NMR spectrum of **6f.**

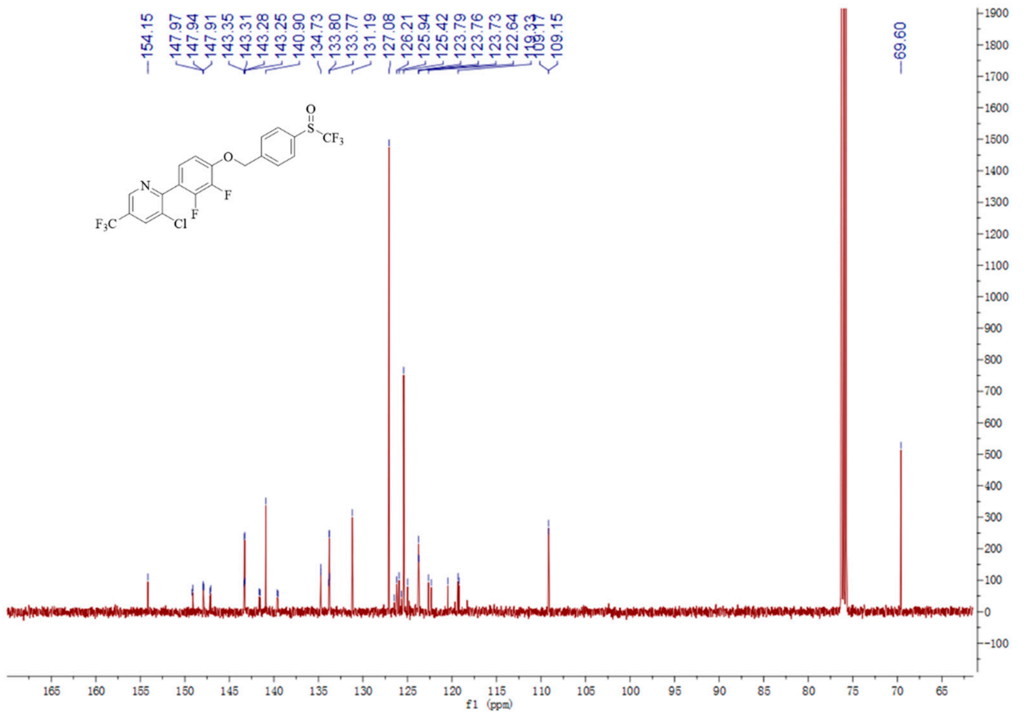

Figure S24. The ^{13}C NMR spectrum of **6f.**

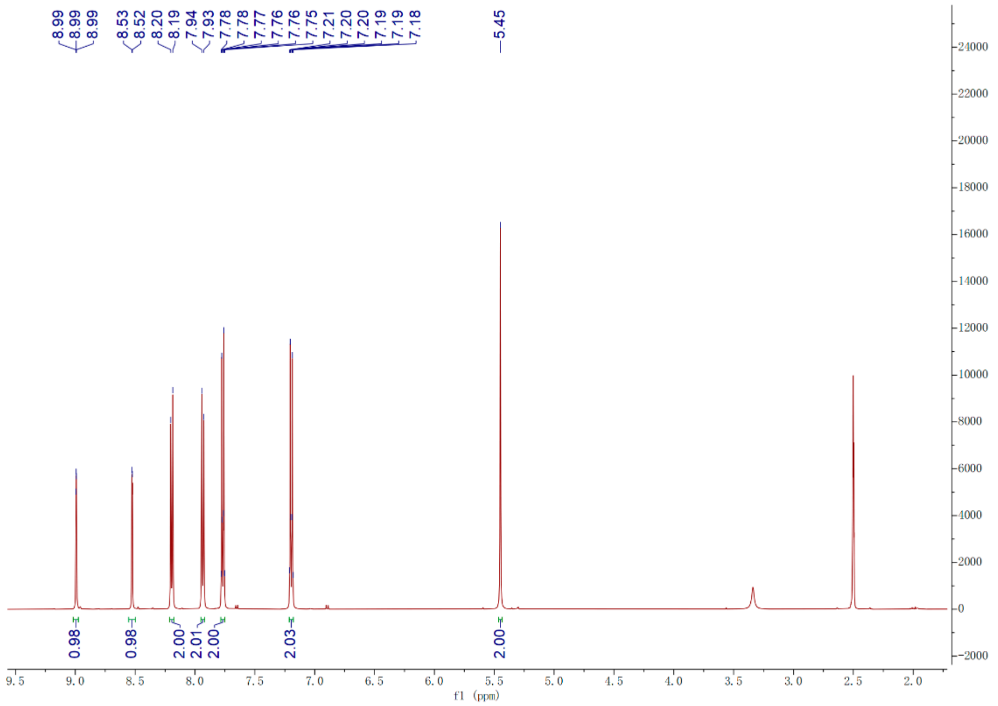

Figure S25. The ^{1}H NMR spectrum of **7a.**

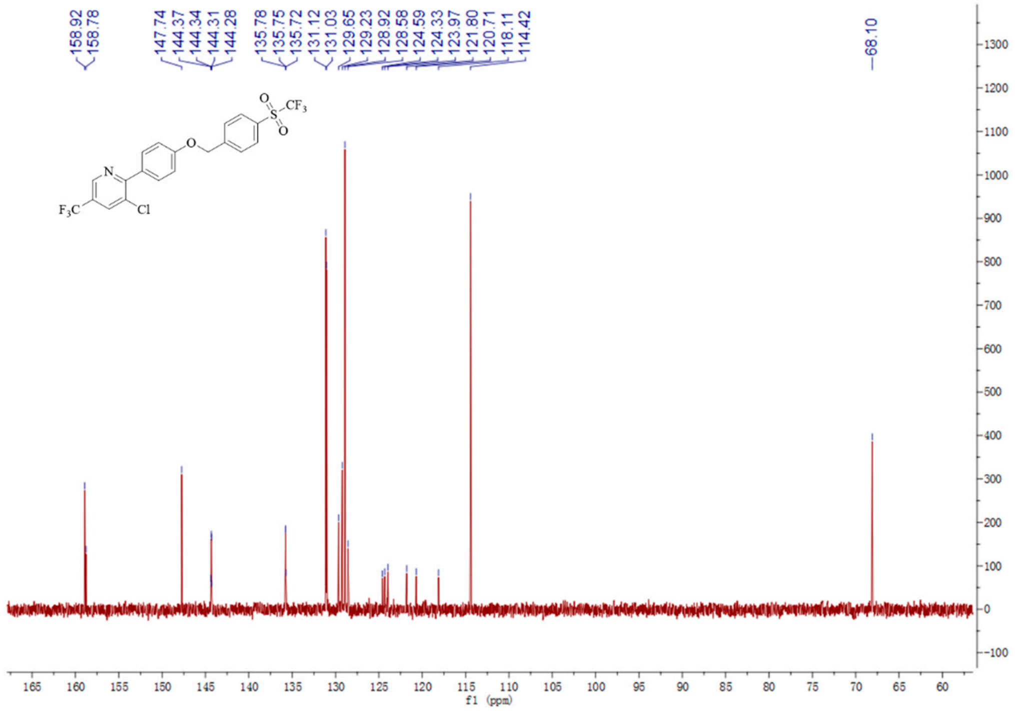

Figure S26. The ^{13}C NMR spectrum of **7a.**

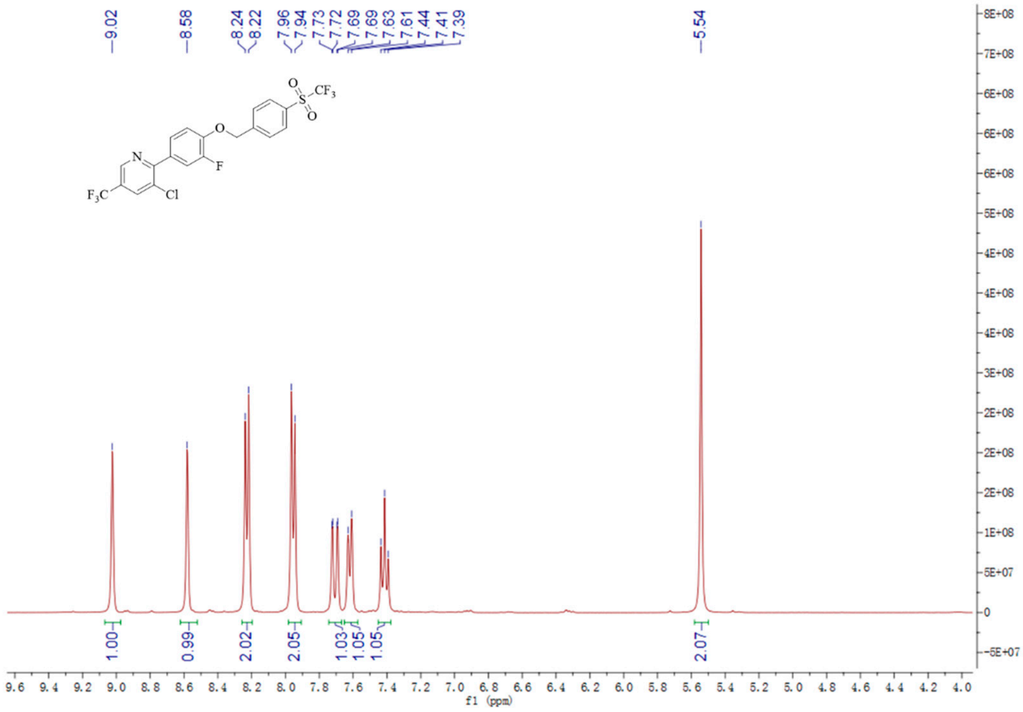

Figure S27. The ^{1}H NMR spectrum of **7b.**

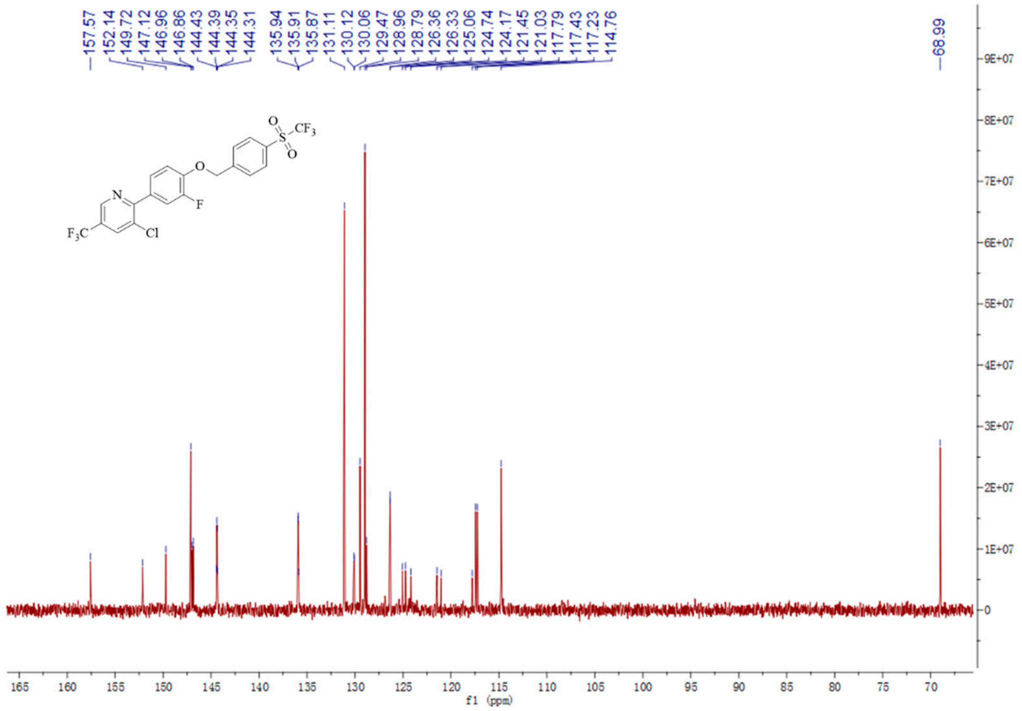

Figure S28. The ^{13}C NMR spectrum of **7b.**

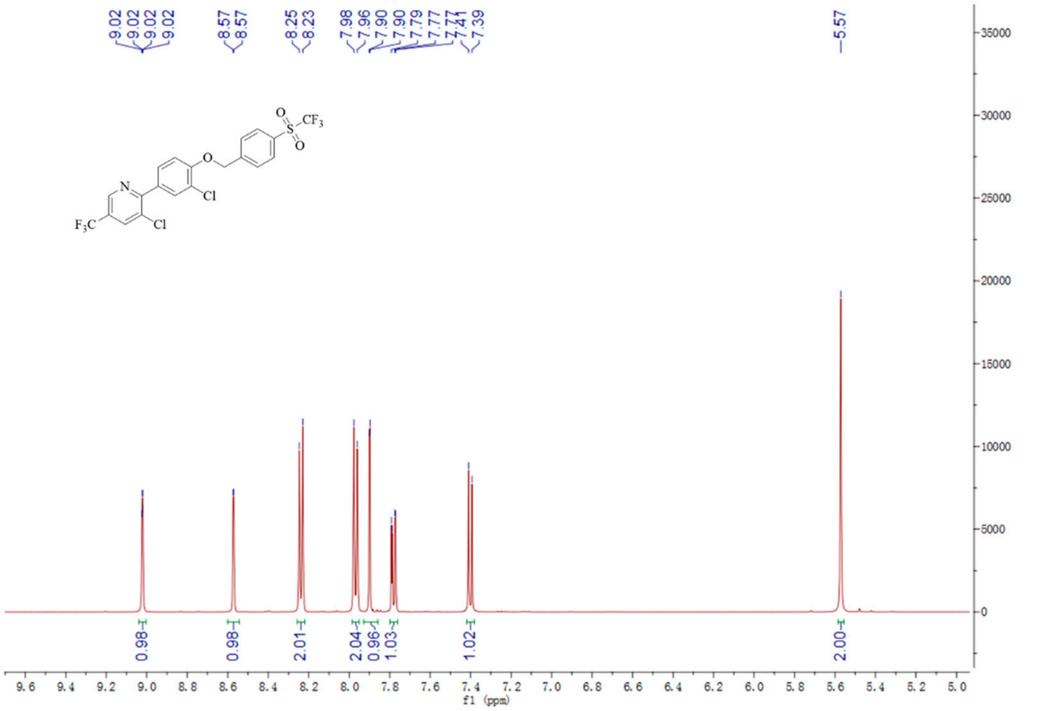

Figure S29. The ^{1}H NMR spectrum of **7c.**

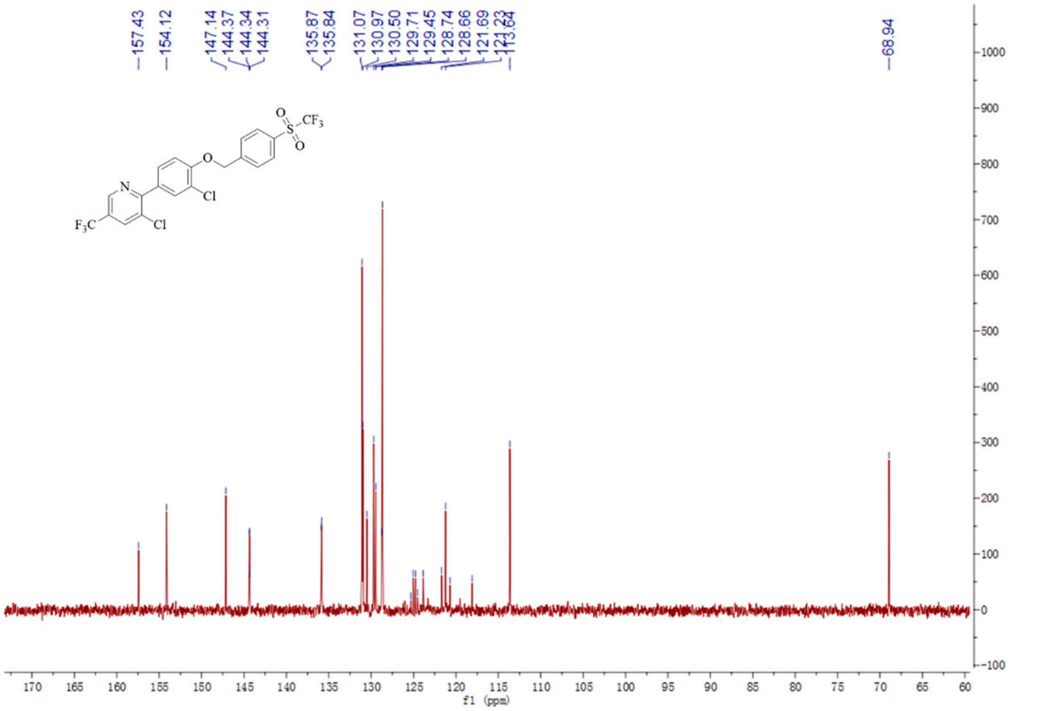

Figure S30. The ^{13}C NMR spectrum of **7c.**

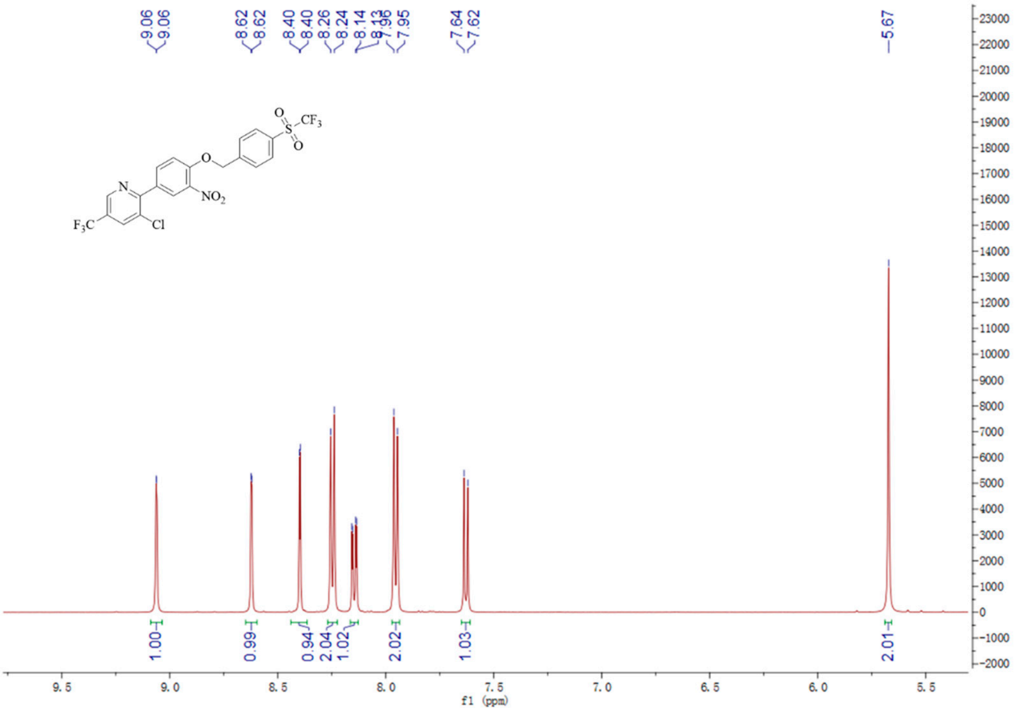

Figure S31. The ^{1}H NMR spectrum of **7d.**

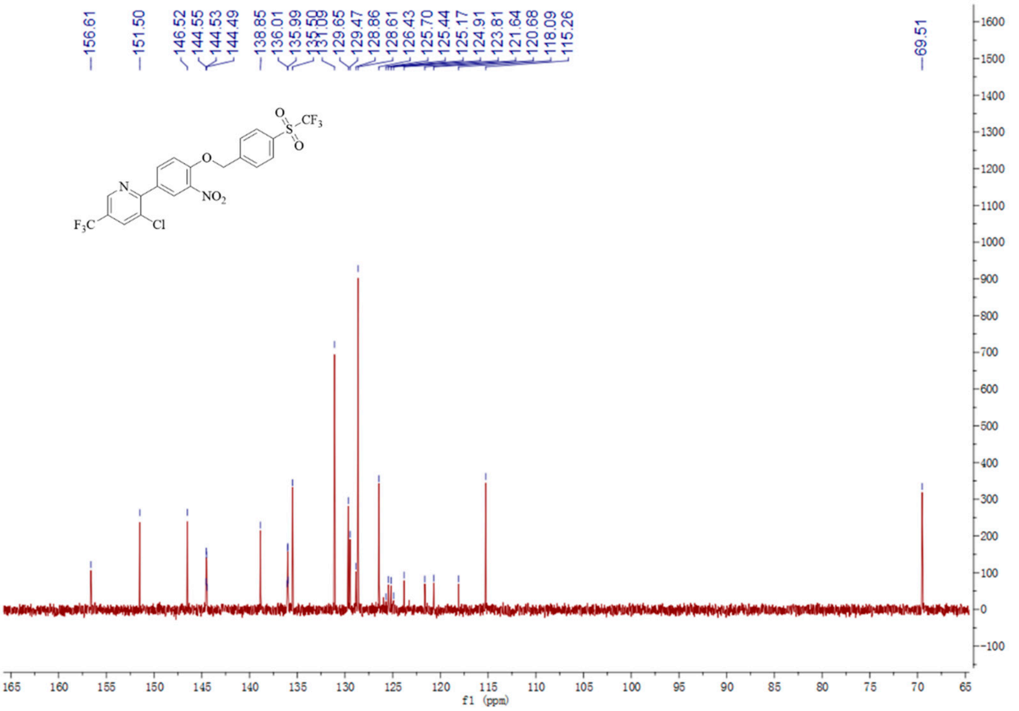

Figure S32. The ^{13}C NMR spectrum of **7d.**

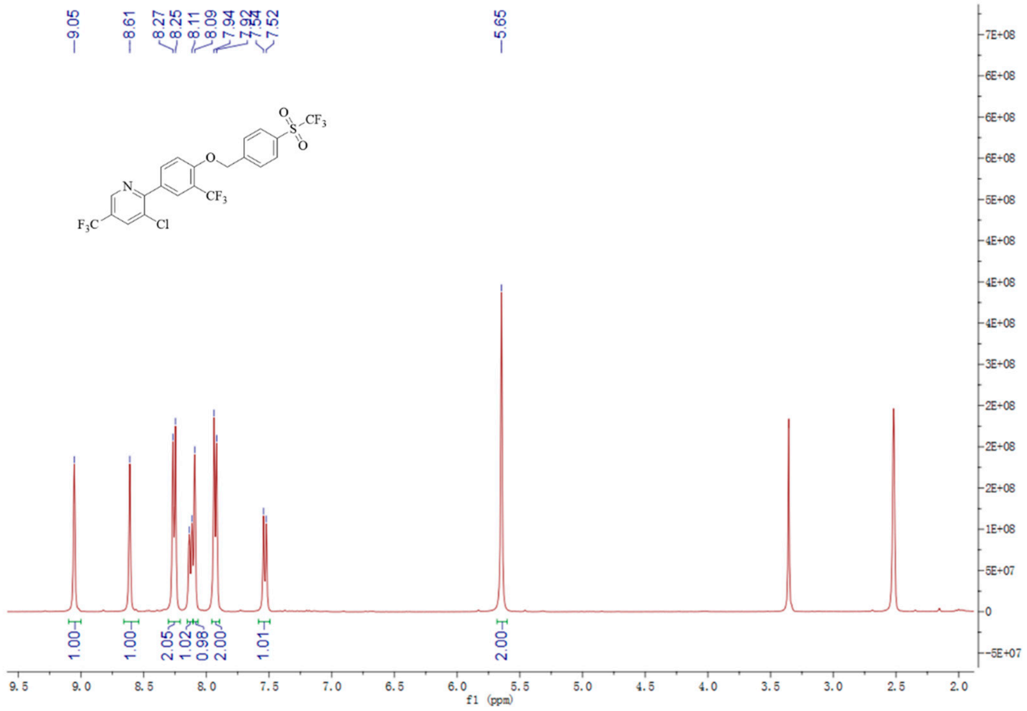

Figure S33. The ^{1}H NMR spectrum of **7e.**

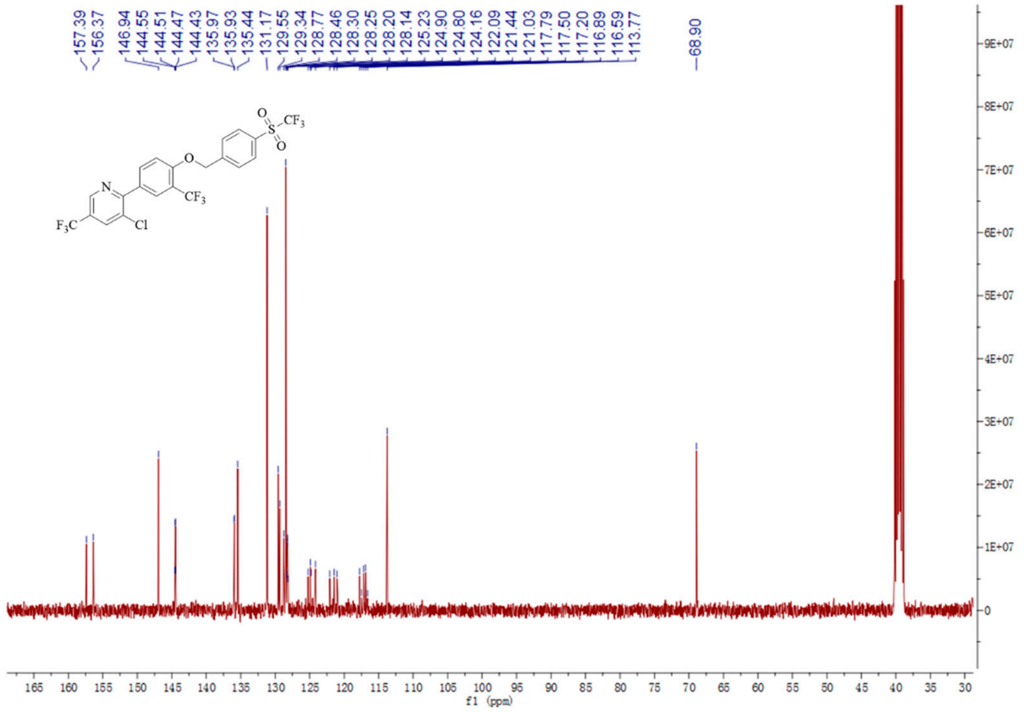

Figure S34. The ^{13}C NMR spectrum of **7e.**

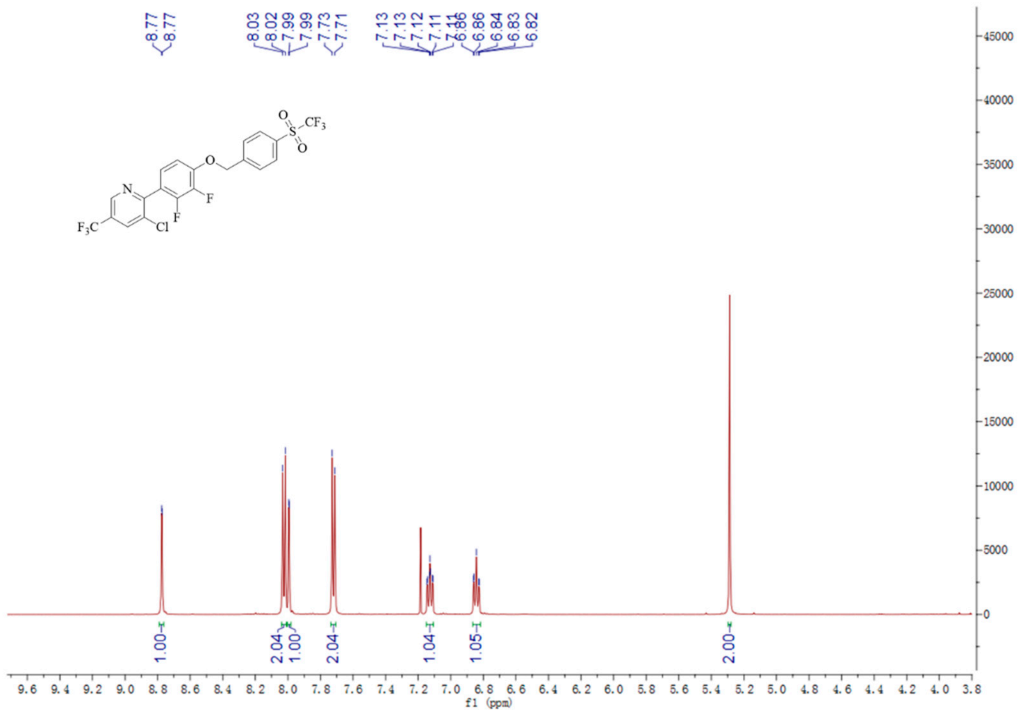

Figure S35. The ^{1}H NMR spectrum of **7f.**

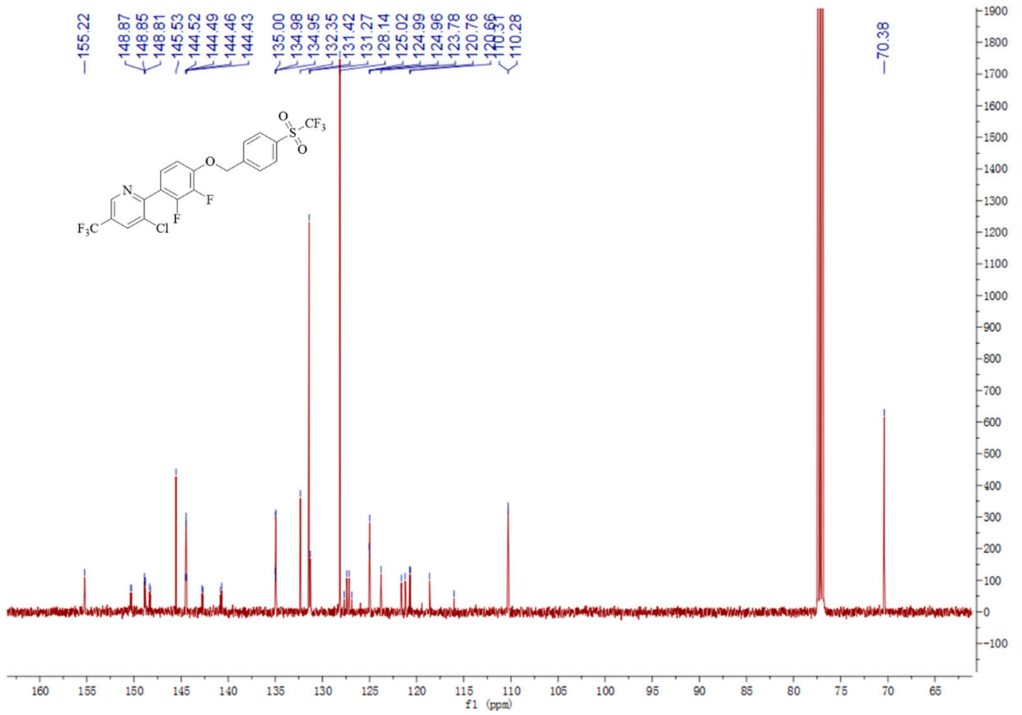

Figure S36. The ^{13}C NMR spectrum of **7f.**
